# Sequential Biologic, Small-Molecule, and GLP-1 Analogue Therapy in Postcolectomy Enteritis With High Output Stoma

**DOI:** 10.14309/crj.0000000000002233

**Published:** 2026-07-06

**Authors:** Zachary Tuttle, Emily Wright, Mark Lust, Cori Behrenbruch, Penelope McKelvie, Ola Niewiadomski

**Affiliations:** 1 Department of Gastroenterology, St Vincent's Hospital, Melbourne, Australia; 2 University of Melbourne, Melbourne, Australia; 3 Department of Colorectal Surgery, St Vincent's Hospital, Melbourne, Australia; 4 Department of Anatomical Pathology, St Vincent's Hospital, Melbourne, Australia

**Keywords:** post-colectomy enteritis, ulcerative colitis, ustekinumab, glucagon-like peptide-1 analogue, high-output stoma

## Abstract

Postcolectomy enteritis is a rare but serious complication after colectomy for ulcerative colitis. We report the case of a 71-year-old man with acute severe ulcerative colitis who developed steroid-refractory postcolectomy enteritis after subtotal colectomy with end ileostomy. Postoperatively, he experienced persistent high output stoma and ongoing ileal inflammation despite treatment with corticosteroids, infliximab, upadacitinib, and octreotide. Induction therapy with ustekinumab led to gradual endoscopic, histological, and radiological improvement. Off-label use of a glucagon-like peptide-1 analogue was initiated for the management of high output stoma, with subsequent reduction in stoma losses and successful weaning from parenteral nutrition.

## INTRODUCTION

Acute severe ulcerative colitis is a life-threatening manifestation of ulcerative colitis (UC), for which colectomy is indicated in medically refractory disease.^1^ Postcolectomy enteritis (PCE) is a rare but serious complication with ill-defined treatment options.^2,3^ We present a case of steroid-refractory PCE managed with sequential biologic and small-molecule therapy, with the use of a glucagon-like peptide-1 (GLP-1) analogue for high output stoma (HOS).

## CASE REPORT

A 71-year-old man with recent diagnosis of UC (Mayo 3 pan-colitis) was referred to hospital with 3 weeks of bloody bowel frequency (18 stools/day). Before admission, he was on oral mesalazine 4 g daily and prednisolone tapered from 40 mg daily. On presentation to the emergency department, vitals were in normal range. Investigations demonstrated a C-reactive protein (CRP) of 70 mg/L, hemoglobin of 168 g/L, platelets of 421 × 10^9^/L, and albumin of 38 g/L. Acute severe ulcerative colitis was diagnosed and intravenous (IV) hydrocortisone 100 mg 4 times a day commenced. Initial workup included stool cultures and *Clostridioides difficile* PCR (negative). Fecal calprotectin was elevated at 1,070 μg/g. Flexible sigmoidoscopy confirmed moderate colitis (Mayo 2) with histology demonstrating moderately active UC, negative for cytomegalovirus. By day 3, he had persistent bloody bowels (12 stools/day) with CRP of 37 mg/L, albumin of 33 g/L, and CRP to albumin ratio (CAR) of 1.121, with subsequent salvage infliximab (IFX) 5 mg/kg initiated, with additional doses on days 6 (5 mg/kg) and 9 (10 mg/kg). Endoscopy demonstrated progressive severe colitis to the point of insertion (proximal sigmoid) with distal rectum mucosal ulceration (Mayo 3). On day 12, he passed 20 bloody stools, with CRP of 25 mg/L, albumin of 24 g/L and CAR of 1.042. Upadacitinib 45 mg daily was introduced off-label as second line salvage therapy. Bloody bowel motions persisted (>10 stools/day) and fecal calprotectin remained high at 2000 μg/g with some improvement systemically (CRP of 7 mg/L) but an albumin of 27 g/L and CAR of 0.259. With inadequate response, upadacitinib was ceased and he underwent subtotal colectomy with end ileostomy on day 18.

**Figure 1.**
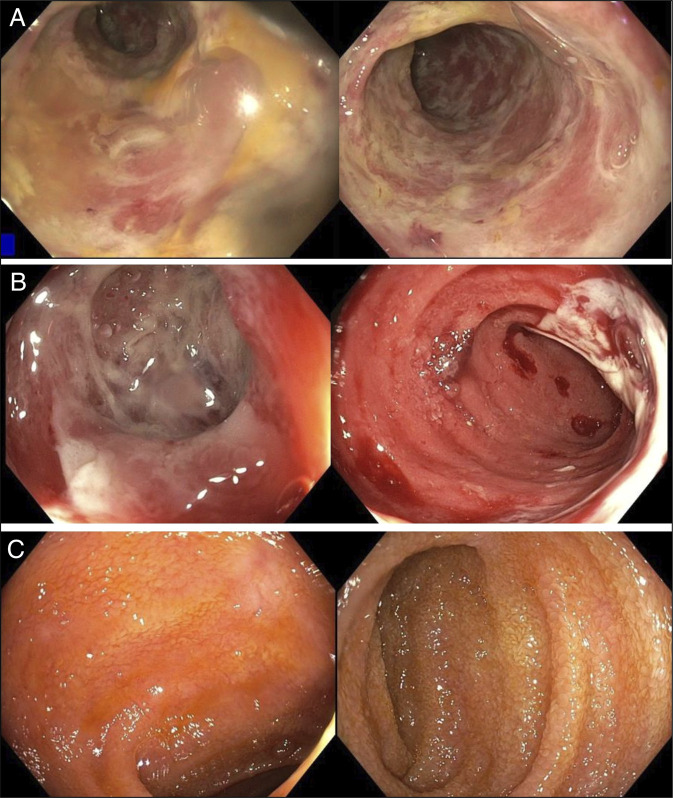
(A) Ileoscopy day 65: scope through ileostomy to 20 cm. Inflamed mucosa with pseudomembranes, very friable. (B) Ileoscopy day 79: scope through ileostomy to 15 cm. Severe ileitis at 5 cm with erythema and friable mucosa. Mild inflammation more proximally. (C) Ileoscopy 7 months post discharge: scope through ileostomy to 30 cm. Terminal ileum appeared normal. Very mild inflammation at the ileostomy inlet.

**Figure 2.**
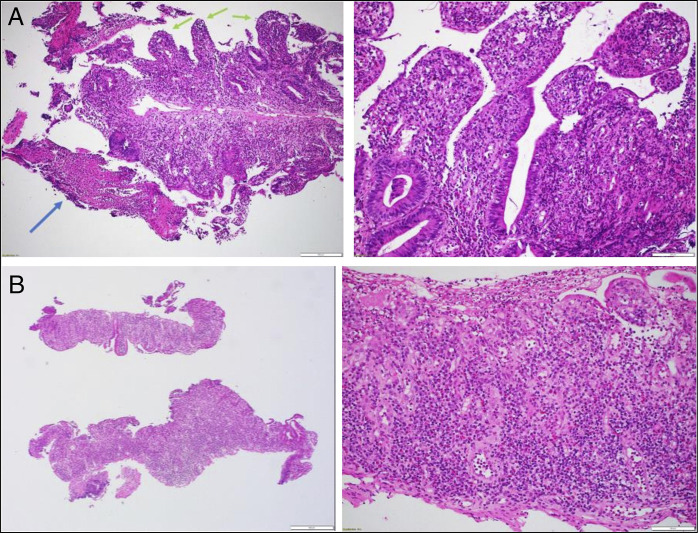
A) Top left (×100): Abnormal ileal mucosa with fibrinopurulent exudate (blue arrow), blunted villi lacking surface epithelium (green arrows), and dense active chronic inflammatory infiltrate within the lamina propria. Top right (×200): Higher magnification demonstrating abnormal villi with mixed inflammatory cell infiltrate. B) Bottom left (×40): Flat ileal mucosa with marked villous atrophy and dense active chronic inflammatory infiltrate within the lamina propria. Bottom right (×200): Higher magnification of flat ileal mucosa showing dense active chronic inflammatory infiltrate within the lamina propria.

**Figure 3.**
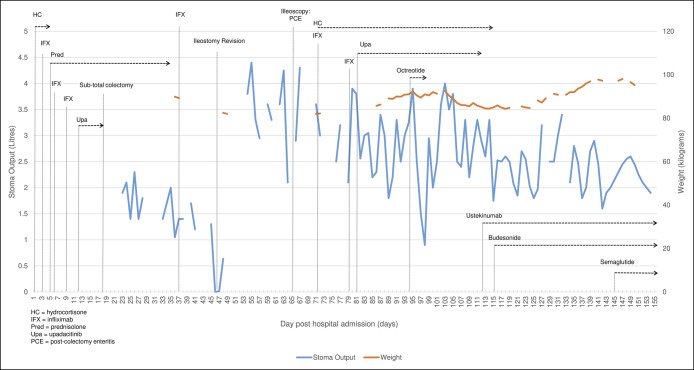
Timeline of key hospital milestones, treatments, and corresponding stoma output and weight.

Stoma output increased to 2.5 L/day by day 4 postcolectomy. Despite standard treatment—limiting hypotonic fluids, anti-motility/secretory therapy and oral rehydration solution—output remained >2 L/day. He also reported rectal stump mucous and bloody discharge. Flexible sigmoidoscopy revealed colitis of the stump (Mayo 2). On day 37, IFX (10 mg/kg) was retrialled, with initial reduction in output (<1.5 L/day) but no change to stump discharge. He subsequently developed small bowel obstruction, necessitating a return to theatre for reduction and decompression of a mesenteric twist. The ileostomy was revised and rectal stump shortened. After surgery, rectal discharge ceased but stoma output remained high (>3 L/day) and total parenteral nutrition (TPN) commenced. Computed tomography abdomen/pelvis revealed long-segment enteritis extending to the ileostomy. Ileoscopy demonstrated severely ulcerated friable mucosa to point of insertion to 20 cm (Figure 1), and histology was consistent with severe active chronic inflammation without viral inclusions and cytomegalovirus negative (Figure 2). The findings were consistent with PCE. On day 71, IFX 10 mg/kg and hydrocortisone 100 mg twice daily were commenced for PCE. Repeat ileoscopy to 15 cm showed ongoing severe ileitis with friable mucosa at 5 cm and mild proximal improvement (Figure 1). Histology confirmed severe enteritis (Figure 2). Furthermore, IFX 10 mg/kg (day 79) was administered without improvement and upadacitinib 45 mg daily commenced (day 83). Stoma output remained high (>3 L/day) and octreotide was trialled with no benefit. Ileoscopy showed moderately severe ileal inflammation and contact bleeding at the stoma site. Given treatment failure, upadacitinib was discontinued and IV ustekinumab 520 mg given on day 112. Magnetic resonance enterography revealed improvement of enteritis, but output remained high (2.5 L/day). On day 145, off-label semaglutide 0.5 mg weekly was commenced for HOS, escalated to 0.75 mg weekly 7 days later. This was associated with mild reduction in output (<2.5–3 L/day) (Figure 3). Home TPN (nightly) and IV fluids was organized, and the patient discharged on day 155 on pantoprazole 40 mg twice daily, codeine 30 mg 4 times a day, loperamide 8 mg 4 times a day, ustekinumab 90 mg eight-weekly, semaglutide 0.75 mg weekly, and weaning budesonide.

At 1 month post-discharge, stoma output declined to <2 L/day and TPN reduced to alternate-day infusions with intermittent IV fluids. By 2 months, stoma output remained <2 L/day and TPN discontinued. At 3 months, stoma output was ∼1.5 L/day and semaglutide ceased. At 6 months, stoma output remained <1.5 L/day and IV fluids ceased. At 7 months, he underwent repeat ileoscopy with normal terminal ileum and mild inflammation at the ileostomy inlet, with nil histological evidence of inflammation (Figure 1).

## DISCUSSION

PCE is an uncommon yet clinically significant entity with life-threatening complications that include perforation, massive bleeding and shock.^4,5^ The rarity and heterogeneity of presentations delay early diagnosis and management. Corticosteroids are regarded as first-line therapy in PCE.^6^ Beyond corticosteroids, tumor necrosis factor inhibitors have been reported as effective.^7,8^ Ustekinumab has been reported once in PCE, trialled fourth line after sequential therapy including hydrocortisone, 3 IFX infusions (5 mg/kg), and cyclosporine A (50 mg). Clinical improvement, including reduction in stoma output, was observed 20 days post-ustekinumab commenced at 4-weekly intervals, with complete mucosal healing at 12 months and resolution of HOS.^9^ Upadacitinib has been reported once in severe Crohn's-like enteritis postcolectomy for UC, although the eventual diagnosis of Crohn's enteritis limits comparison with our case and differs from the typical presentation of PCE.^10^ To the best of our knowledge, this is the first report of sequential advanced therapies in PCE, involving treatment with upadacitinib followed by ustekinumab. The off-label use of GLP-1 analogue was due to the restricted availability of GLP-2 analogues in Australia and emerging evidence supporting its use in HOS.^11^ Interpretation of the benefit of GLP-1 analogue therapy is limited by its temporal overlap with ustekinumab, precluding definitive attribution of the clinical response. IFX level monitoring was not performed, limiting interpretation of the apparent treatment failure.

Our case illustrates the safety of sequential use of biologic and small-molecule therapies with successful response to ustekinumab. In addition, we report the novel use of a GLP-1 analogue to mitigate persistent HOS.

## DISCLOSURES

Author contributions: Z. Tuttle conducted the literature search, drafted the manuscript and synthesized the data. ON contributed to study conception and critically reviewed the manuscript. O. Niewiadomski, E. Wright, M. Lust, C. Behrenbruch, and P. McKelvie were involved in the management of the case. All authors approved the final version and take responsibility for its content. Z. Tuttle is the article guarantor.

Financial disclosure: None to report.

Informed consent was obtained for this case report.

## ABBREVIATIONS:

ASUC: acute severe ulcerative colitis
CAR: C-reactive protein-to-albumin ratio
CRP: C-reactive protein
FC: fecal calprotectin
GLP-1: glucagon-like peptide-1
HOS: high-output stoma
IFX: infliximab
IV: intravenous
ORS: oral rehydration solution
PCE: post-colectomy enteritis
PCR: polymerase chain reaction
QID: four times daily
TNF: tumor necrosis factor
TPN: total parenteral nutrition
UC: ulcerative colitis

## References

[1] Calméjane L Laharie D Kirchgesner J Uzzan M. Review article: Updated management of acute severe ulcerative colitis: From steroids to novel medical strategies. United Eur Gastroenterol J. 2023;11(8):722–32. 10.1002/ueg2.12442 PMC10576604 37475143

[2] Horio Y Uchino M Hori K . Clinical features and therapeutic outcomes of post-colectomy enteritis with ulcerative colitis. J Anus Rectum Colon. 2021;5(4):405–13. 34746505 10.23922/jarc.2021-031 PMC8553349

[3] Gonella F Massucco P Daperno M . Ulcerative enteritis. How the extension of ulcerative colitis to small bowel may jeopardize postcolectomy course: A case report and literature review. Eur J Gastroenterol Hepatol. 2021;33(4):589–94. 33657604 10.1097/MEG.0000000000002112

[4] Annese V Caruso N Bisceglia M . Fatal ulcerative panenteritis following colectomy in a patient with ulcerative colitis. Dig Dis Sci. 1999;44(6):1189–95. 10389695 10.1023/a:1026688526551

[5] Toritani K Kimura H Maebashi M . Massive bleeding and perforation due to post-colectomy pan-enteritis with a significant response to biologic in a patient with ulcerative colitis: A case report. Surg Case Rep. 2024;10(1):201. 39196494 10.1186/s40792-024-02003-8 PMC11358364

[6] Truelove SC Witts LJ. Cortisone in ulcerative colitis; final report on a therapeutic trial. Br Med J. 1955;2(4947):1041–8. 13260656 10.1136/bmj.2.4947.1041 PMC1981500

[7] Rush B Berger L Rosenfeld G Bressler B. Tacrolimus therapy for ulcerative colitis-associated post-colectomy enteritis. ACG Case Rep J. 2014;2(1):33–5. 26157899 10.14309/crj.2014.76 PMC4435352

[8] Shimoda F Kuroha M Chiba H . Ulcerative colitis-related postoperative enteritis treated with anti-tumor necrosis factor therapy: Two case reports and a literature review. Clin J Gastroenterol. 2021;14(5):1396–403. 34302278 10.1007/s12328-021-01485-5

[9] Guo C He S Wang H. Case report: Successful treatment of ulcerative colitis-related post-colectomy enteritis refractory to multiple therapies with ustekinumab. Front Immunol. 2024;15:1297508. 38433841 10.3389/fimmu.2024.1297508 PMC10904456

[10] Chan L Lam E Leung Y Chen Kiow JL. S3918 Upadacitinib for severe Crohn's-like enteritis following colectomy for ulcerative colitis. Am J Gastroenterol. 2024;119(10S):S2551–2.

[11] Merlo FD Aimasso U Ossola M . Effects of treatment with liraglutide early after surgical intervention on clinical outcomes in patients with short bowel syndrome: A pilot observational “real-life” study. Nutrients. 2023;15(12):2740. 37375644 10.3390/nu15122740 PMC10305110

